# Plant defense and herbivore counter-defense: benzoxazinoids and insect herbivores

**DOI:** 10.1007/s11101-016-9481-1

**Published:** 2016-11-05

**Authors:** Felipe C. Wouters, Blair Blanchette, Jonathan Gershenzon, Daniel G. Vassão

**Affiliations:** Department of Biochemistry, Max Planck Institute for Chemical Ecology, Hans-Knöll-Str. 8, 07745 Jena, Germany

**Keywords:** Chemical ecology, Detoxification, Nutritional indices, Metabolism, Poaceae

## Abstract

Benzoxazinoids are a class of indole-derived plant chemical defenses comprising compounds with a 2-hydroxy-2*H*-1,4-benzoxazin-3(4*H*)-one skeleton and their derivatives. These phytochemicals are widespread in grasses, including important cereal crops such as maize, wheat and rye, as well as a few dicot species, and display a wide range of antifeedant, insecticidal, antimicrobial, and allelopathic activities. Although their overall effects against insect herbivores are frequently reported, much less is known about how their modes of action specifically influence insect physiology. The present review summarizes the biological activities of benzoxazinoids on chewing, piercing-sucking, and root insect herbivores. We show how within-plant distribution modulates the exposure of different herbivore feeding guilds to these defenses, and how benzoxazinoids may act as toxins, feeding deterrents and digestibility-reducing compounds under different conditions. In addition, recent results on the metabolism of benzoxazinoids by insects and their consequences for plant-herbivore interactions are addressed, as well as directions for future research.

## Introduction

**Fig. 1:**
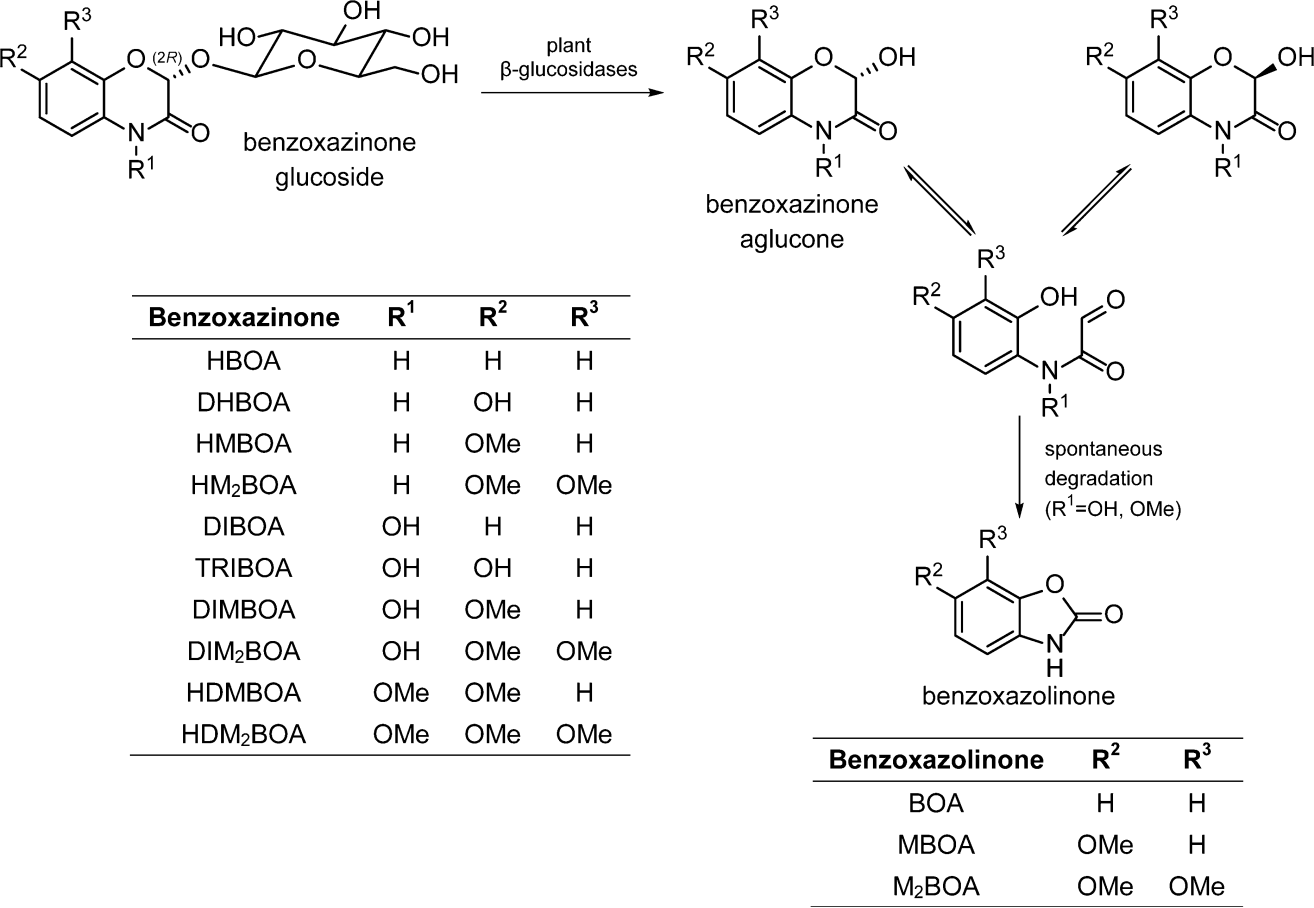
Glucoside hydrolysis of naturally occurring benzoxazinones and degradation to benzoxazolinones via oxo-cyclo/ring-chain tautomerism

Plants have evolved a diverse repertoire of specialized or “secondary” metabolites in order to alleviate biotic and abiotic stresses. Among these, benzoxazinoids are a group of important defense chemicals widespread in grasses (Poaceae), including economically important crops such as maize, wheat, and rye (but not rice, oat, sorghum, and cultivated barley) (Niemeyer 2009). This class of compounds is also produced by individual species within the dicot families Acanthaceae, Ranunculaceae, Plantaginaceae, and Lamiaceae (Frey et al. 2009; Makowska et al. 2015). These indole-derived compounds are regarded as general defense metabolites in plants, being associated with a wide spectrum of direct antifeedant, insecticidal, antimicrobial, and allelopathic activities (Niemeyer 2009), as well as serving to regulate other defense mechanisms (Maag et al. 2015a). Due to their structural diversity, the term benzoxazinoids (BXDs) will be used in this article to refer to both benzoxazinones (glucosides and corresponding aglucones containing a 2-hydroxy-2*H*-1,4-benzoxazin-3(4*H*)-one skeleton) and their degradation products, benzoxazolinones. The most common naturally occurring BXD structures, simplified activation and degradation routes, and commonly used acronyms are presented in Fig. 1.

Benzoxazinones are commonly assumed to be stored as glucosides in vacuoles of undamaged plant cells, and hydrolysis by β-glucosidases increases their reactivity and toxicity (Frey et al. 2009; Niemeyer 2009). BXD-hydrolyzing glucosidases can be found in plastids, cytoplasm, and cell walls (i.e. spatially separated from their glucoside substrates) (Nikus et al. 2001) which upon damage to the plant cell and loss of tissue and cell integrity catalyze the hydrolysis of benzoxazinone glucosides. The resulting unstable benzoxazinone aglucones and their benzoxazolinone degradation products are regarded as the compounds mediating most observed BXD biological activities (Niemeyer 2009; Sicker and Schulz 2002). This compartmentalized system consisting of a stable compound and an activating enzyme resembles other activated two-component defense systems in plants such as glucosinolates and cyanogenic and iridoid glycosides (Morant et al. 2008; Pentzold et al. 2014b).

The effects of a variety of BXDs on the survival, growth and feeding behavior of a range of organisms have been studied for many years. Nevertheless, the underlying molecular modes of action and physiological mechanisms responsible for these effects are not completely understood. In addition, the importance of these chemical defenses in a natural context and their modulation by specific characteristics, such as physiology of the target organism and its feeding behavior, are not usually critically evaluated. Given the tissue-specific distribution of BXDs in plants and their activation by glucosidases, the feeding behavior of insect herbivores dictates the amount and the nature of BXDs that they will be exposed to, based on the attacked tissue and the extent of plant cell damage. Finally, even after ingestion of BXDs by the target organism, specialized metabolism of such compounds, as well as other biochemical strategies to avoid toxicity, might play a role on their overall biological effects.

Due to the economic and ecological importance of BXDs, many aspects of these compounds have been reviewed, including their biological activities (Macías et al. 2009; Niemeyer 2009), organic synthesis (Macías et al. 2006; Sicker and Schulz 2002), chemical reactivities (Hashimoto and Shudo 1996; Wouters et al. 2016), and biosynthetic evolution and genetics (Frey et al. 2009; Makowska et al. 2015). The present review aims to categorize the spectrum of biological activities observed for BXDs towards insect herbivores with different feeding behaviors: chewing, piercing-sucking, and root herbivores. These effects are discussed in the ecological context of each interaction, and the contribution of toxicity, digestibility-reduction and antifeedant activities to the overall effects observed on insect herbivores is addressed. Additionally, current knowledge about the metabolism of BXDs in various organisms is summarized. We begin with overviews of BXD biosynthesis, plant distribution and chemical properties to put the biological activities in the proper context.

## Biosynthesis and distribution of BXDs in plants

**Fig. 2:**
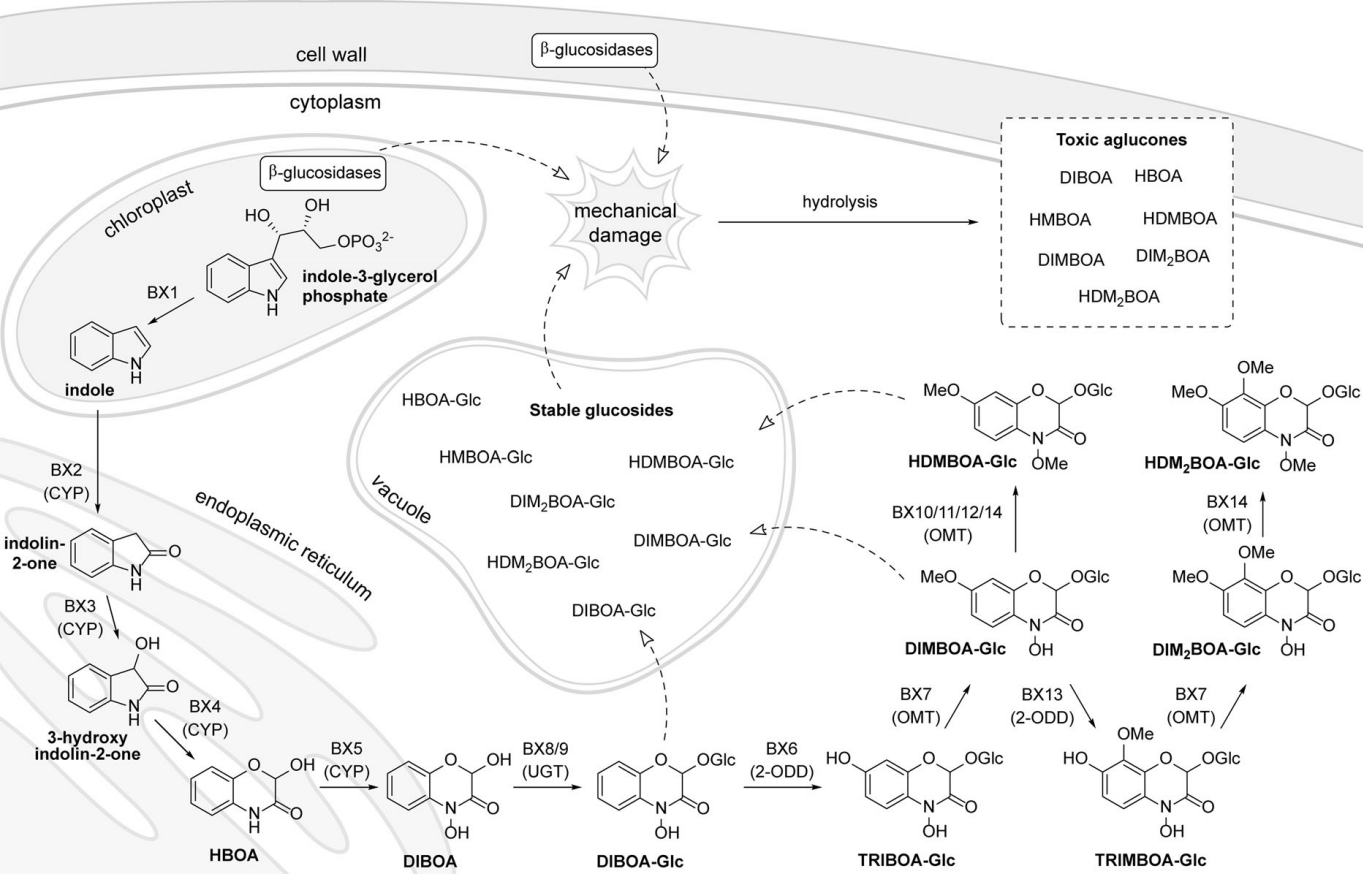
BXD biosynthesis and compartmentalization in plant cell

The BXD biosynthetic pathway has been mostly established in maize (Frey et al. 2009; Meihls et al. 2013), but other BXD-producing plants have also been investigated (Dick et al. 2012; Schullehner et al. 2008). The general reactions and compartmentalization of BXD biosynthesis are shown in Fig. 2. The formation of BXDs, as well as their genetics and evolution in plants have been comprehensively reviewed (Frey et al. 2009; Gierl and Frey 2001; Makowska et al. 2015).

The first committed step of the pathway is catalyzed by BX1, which converts indole-3-glycerol phosphate into indole in the chloroplasts. This enzyme is a homolog of the α-subunit of tryptophan synthase (TSA). In tryptophan synthase, the resulting indole is not released, but rather channeled to the active site of a β subunit (TSB), where it reacts with serine, yielding tryptophan and water. Most likely, the gene encoding BX1 originated from that encoding TSA by duplication and modification of both function and expression patterns. These changes gave rise to both *Bx1* and *Igl* (indole-3-glycerol phosphate lyase), which mostly produces the free indole released by the plant as a volatile (Gierl and Frey 2001). After this first step, free indole is converted to DIBOA by incorporation of four oxygen atoms. These oxidations are carried out by four cytochrome P450-dependent monooxygenases, BX2-BX5, which are located in the endoplasmic reticulum and are substrate-specific and regioselective for the sequential introduction of oxygen atoms. The DIBOA aglucone thus produced is rendered less reactive in the cytoplasm via glucosylation by the action of the UDP-glucosyltransferases (UGTs) BX8 and BX9, providing a more stable intermediate for further modifications. DIBOA-Glc can be then hydroxylated by the 2-oxoglutarate-dependent dioxygenase BX6, and further *O*-methylated by the *O*-methyltransferase BX7, forming DIMBOA-Glc. HDMBOA-Glc can then be formed from DIMBOA-Glc via an *O*-methylation reaction catalyzed by a group of three homologous *O*-methyltransferases, BX10, BX11, and BX12 (formerly named BX10a, BX10b, and BX10c, respectively, in Meihls et al. 2013). Recently, an additional branch in the BXD pathway has been described: BX13, a 2-oxoglutarate-dependent dioxygenase, catalyzes the conversion of DIMBOA-Glc to TRIMBOA-Glc; the latter can be *O*-methylated by BX7 to form DIM_2_BOA-Glc, which can be further methylated by the *O*-methyltransferase BX14 to generate HDM_2_BOA-Glc (Handrick et al. 2016).

The stable BXD glucosides are considered to be transported and stored in the vacuole, while BXD-hydrolyzing β-glucosidases are thought to be present in plastids (Frey et al. 2009; Niemeyer 2009; Sicker and Schulz 2002). However, this distribution is not well established and may vary with plant species, tissue and age. Some direct evidence of BXD glucoside distribution was provided by MALDI-MS imaging of metabolites in a maize leaf cross-section, which revealed that DIMBOA-Glc and HMBOA-Glc were localized in cell vacuoles (Korte et al. 2015). The use of antibodies suggests that the subcellular distribution of β-glucosidases varies among plant species and tissues (Nikus et al. 2001). Wheat and rye β-glucosidases are mainly localized in cell walls and cytoplasm, while in maize they are mostly found in plastids and proplastids. However, deviations from these general trends have been noted. For instance, Massardo et al. (1994) observed through cell fractionation the presence of BXD β-glucosidases in the vacuole and DIMBOA-Glc in the extravacuolar space of maize parenchyma cells.

Upon destruction of the tissue and its accompanying cellular organization caused for example by herbivore damage or pathogen attack, the stable glucosides come into contact with β-glucosidases and are hydrolyzed to reactive aglucones, which are then implicated in BXD toxicity (Cambier et al. 1999). But, in some cases aglucones accumulate. For example, the apoplast of maize leaves contains DIMBOA as well as DIMBOA-Glc and HDMBOA-Glc (Ahmad et al. 2011), while waxes on the surface of maize whorls contain considerable amounts of HDMBOA aglucone (which seems to be stable in the waxy layer), together with DIMBOA and MBOA (Hedin 1993). In roots, BXD aglucones are considered to be actively exuded and diffuse into the soil where they exert their effects on soil microorganisms, root herbivores, and other plants (Belz and Hurle 2005; Pérez and Ormeño-Nuñez 1991). The identity of BXDs exuded by roots seems to vary according to growing conditions, sampling, and analytical methods. The aglucones DIBOA and DIMBOA were the main BXDs exuded from roots of hydroponically grown *Secale cereale* and three *Triticum* species: *T. aestivum*, *T. durum*, and *T. spelta* (Belz and Hurle 2005). Maize root exudates collected by a trapping system contained the hydroxamic acids DIMBOA and DIBOA, the lactam HMBOA, and the benzoxazolinones MBOA and BOA. In maize hydroponic cultures, however, the glucoside DIMBOA-Glc was additionally detected (Friebe et al. 1998). Exudate extracts obtained by dipping maize roots in dichloromethane contained mainly the unstable derivative HDMBOA (Zhang et al. 2000). However, a method based on liquid extraction surface analysis detected glucosides such as HDMBOA-Glc and DIMBOA-Glc in maize root exudates (Robert et al. 2012).

The abundance of BXDs and their proportions vary between plant species and varieties, and also among tissues and developmental stages within plants. For example, the main BXD in rye is DIBOA-Glc (Oikawa et al. 2002), whereas DIMBOA-Glc is the major BXD in aerial parts of wheat and maize (Cambier et al. 2000; Oikawa et al. 2002). In contrast, HDMBOA-Glc is dominant in maize roots, with BXDs being more concentrated in crown roots than in primary and secondary roots (Robert et al. 2012). In maize, BXDs reach the highest concentrations in seedlings that are 10 days-old and decline as the plant grows further (Cambier et al. 2000). Moreover, BXDs are differently allocated in leaves according to their age: DIMBOA-Glc was the predominant BXD in young and old maize leaves on plants at growth stages L2 to L4, but at stages L5 to L7 DIBOA-Glc and HMBOA-Glc became the most abundant in older, but not younger leaves (Köhler et al. 2015).

The total concentrations of BXDs in different plants can vary with age and biotic stresses, and can reach >0.1 % of maize leaf fresh weight after caterpillar attack (Dafoe et al. 2011, Glauser et al. 2011). HDMBOA-Glc is highly induced in maize after herbivory (Dafoe et al. 2011, Glauser et al. 2011), fungal attack (Oikawa et al. 2004), and in both maize and wheat upon jasmonic acid treatment (Oikawa et al. 2001, 2002). Moreover, young maize leaves display higher inducibility of HDMBOA-Glc and HDM_2_BOA-Glc upon herbivory than old leaves (Köhler et al. 2015). The induction of these two BXDs is highly localized to the feeding site and their levels remain high for several days (Maag et al. 2016). However, changes in BXD levels away from the feeding area within the same leaf are less pronounced, and no changes are detected in unattacked leaves.

## Chemical properties and reactivity of BXDs

Benzoxazinones can be divided according to their substituent group R^1^ as lactams (R^1^ = H), hydroxamic acids (R^1^ = OH), and *N*–*O*-methylated derivatives (R^1^ = OMe) (Fig. 1). These functional groups and other substituents modulate the stability and reactivity of these compounds and therefore their biological activities.

Benzoxazinone glucosides are remarkably stable (Hietala et al. 1960) and require the action of glucosidases for their hydrolysis. The resulting aglucones are cyclic hemiacetals that undergo oxo-cyclo/ring-chain tautomerism via a fast, reversible ring opening reaction (Copaja et al. 1986), and therefore occur as racemic mixtures in solution. However, all known benzoxazinone glucosides produced by plants are (2*R*)-2-β-D-glucosides (Hartenstein et al. 1993; Hartenstein and Sicker 1994; Kluge et al. 1997; Nagao et al. 1985).

The high activity of BXD β-glucosidases and the instability of aglucones represent a challenge in their extraction and quantitative analysis from natural samples. Once plant material is mechanically disrupted, plant β-glucosidases may quickly hydrolyze benzoxazinone glucosides to aglucones, which spontaneously degrade to benzoxazolinones. The extraction procedure and analytical methods for BXDs have been thoroughly evaluated and compared (Villagrasa et al. 2009), and modern LC–MS protocols are sensitive and accurate. It is important to note, however, that earlier experiments sometimes used colorimetric methods to determine total hydroxamic acids, with no distinction between different structures or even between glucosides and aglucones. Other methods have included calculations of DIMBOA content based on degradation to MBOA, which can be inaccurate due to the non-quantitative nature of this transformation (Woodward et al. 1978) and the fact that HDMBOA also degrades to MBOA. In fact, due to its instability, HDMBOA was likely often missed in chemical analyses for many years, only being considered as a major benzoxazinoid starting in the 1990s (Hedin 1993), although the glucoside was already identified in the 1970s (Hofman et al. 1970). Therefore, when interpreting results in the literature, one must consider possible quantification errors and the fact that certain BXDs were not typically detected.

In general, lactams (N–H compounds) are not degraded to benzoxazolinones, whereas hydroxamic acids (N–OH) degrade readily and *N*–*O*-methyl derivatives degrade even faster than hydroxamic acids. For example, the half-lives of HDMBOA and DIMBOA aglucones in buffered D_2_O at pH 5.5 and 24 °C are 1.8 and 25 h, respectively (Maresh et al. 2006). The degradation rates depend on conditions such as pH, temperature and solvent, and structural features such as the nature of the *N*-substituent group and other substituents on the aromatic ring (Atkinson et al. 1991). Several mechanisms have been proposed to explain this degradation (Bredenberg et al. 1962; Grambow et al. 1986; Maresh et al. 2006; Niemeyer et al. 1982a; Smissman et al. 1972; Wouters et al. 2016).

Due to their unique combination of structural features, BXDs are subject to a range of possible reactions that have biological relevance. Upon ring opening, benzoxazolinone aglucones become α-oxo-aldehydes, which are potent electrophiles capable of reacting with nucleophilic residues of proteins, such as thiols and amines, and causing enzymatic inhibition (Atkinson et al. 1991; Niemeyer et al. 1982b; Pérez and Niemeyer 1989). The nitrogen atom can also be an electrophilic site upon elimination of the *N*-substituent group and resulting formation of a nitrenium ion or a reactive *o*-imidoquinone intermediate (Atkinson et al. 1991; Dixon et al. 2012; Hashimoto et al. 1991; Maresh et al. 2006; Quiroz and Niemeyer 1991). BXD hydroxamic acids possess metal ion chelating properties (Tipton and Buell 1970) that can play a role in Fe(III) uptake (Pethő 1992a, b, 1993, 2002) and Al(III) resistance by roots (Poschenrieder et al. 2005). Furthermore, benzoxazolinones can interfere with auxin binding in plants (Hasegawa et al. 1992; Hoshisakoda et al. 1994; Venis and Watson 1978), and the products of their metabolism by soil microorganisms are suggested to play a role in allelopathy (Kato-Noguchi et al. 2010; Schulz et al. 2013; Venturelli et al. 2015).

## Biological effects of BXDs

Since their discovery, BXDs have been considered to function in the plant as resistance factors against herbivores, pathogens, and other plants. Many in vitro studies and bioassays have demonstrated that these compounds have inhibitory and toxic effects towards a wide range of target enzymes and organisms, particularly insect herbivores. Most reports focus on important pest species of cereal crops, and include insects from different ecological guilds: caterpillars (chewing herbivores), aphids (piercing-sucking herbivores), and rootworms (root herbivores). These studies are summarized in the following sections and discussed in the ecological context of insect feeding behavior. Moreover, the influence of the toxicity, digestibility-reduction, and antifeedant activities of BXDs on insect physiology is addressed. Allelopathic and antimicrobial activities of BXDs will not be covered in this section as they have already been comprehensively discussed in recent reviews (Macías et al. 2009; Niemeyer 2009; Schulz et al. 2013).

BXDs have been shown to be present in whole grain cereals, flours, sprouts, bread and beverages derived from rye, wheat, and maize, and might influence human health. Anti-inflammatory, anti-cancer, and anti-microbial activities, as well as stimulatory effects on the central nervous and reproductive systems have been reported for BXDs, mostly using in vitro studies, and the human therapeutic potential of BXDs has been recently reviewed (Adhikari et al. 2015).

### Effects on chewing herbivores

Due to their feeding behavior, chewing herbivores such as lepidopteran larvae disrupt the compartmentalization of BXDs in plant cells during ingestion and are therefore directly exposed to high amounts of BXD aglucones, especially when feeding on leaves from young seedlings where BXD concentrations are highest. Indeed, DIMBOA and BXDs in general have been long known for their toxic and antifeedant activities on caterpillars and for playing an important role in protecting resistant maize lines from herbivore attack (Klun et al. 1967; Reed et al. 1972; Robinson et al. 1978). When evaluating the effects of BXDs on herbivores, it is essential to take into account that most lepidopterans possess an alkaline gut environment (Berenbaum 1980), which facilitates the degradation of hydroxamic acids and *N*–*O*-methylated hydroxamic acids into benzoxazolinones, thus leading to biological activities that can be specific to these insects. Furthermore, lepidopteran feeding can also lead to induction of plant BXDs. In the next two sections, we summarize the effects of BXDs on two lepidopteran genera for which considerable data are available in an effort to compare the differences between specialist and generalist feeders in performance, physiology, mode of action and feeding preference, as well as to compare the effects of various BXD structures.

#### Genus *Ostrinia*

The toxicity and deterrence of BXDs towards the European corn borer (ECB, *Ostrinia nubilalis*) have been extensively investigated due to its economic importance as a pest of maize. When fed in artificial diet, DIMBOA concentrations between 0.05 and 0.5 mg/g (~0.24 and 2.37 mM, respectively) and MBOA at concentrations of 0.5–4.0 mg/g (~3–24 mM, respectively) both increased mortality and developmental times to pupation in a dose-dependent way, but did not affect total weight gain in larvae up to the fifth instar (Campos et al. 1988, 1989). DIMBOA reduced pupal and adult weights in all tested concentrations, and the highest concentration also increased pupal mortality and delayed the emergence of adults. The effects caused by MBOA were similar to those caused by DIMBOA but required higher concentrations, agreeing with the trend that benzoxazolinones are less toxic than benzoxazinones.

Further information on the structure–activity relationships of various BXDs fed to *O. nubilalis* comes from a study employing artificial diets containing 0.5 mM of individual BXDs (Atkinson et al. 1992). Among the natural BXD structures used, DIMBOA and DIBOA showed the highest toxicity, followed by DIM_2_BOA. The authors suggested that the resulting toxicities are positively linked to the degradation rates of these hydroxamic acids to benzoxazolinones. Indeed, the lactams HMBOA and HBOA, which do not form benzoxazolinones upon degradation, did not inhibit larval growth. Thus degradation of benzoxazinones to benzoxazolinones correlates with increasing toxicity although the benzoxazolinones themselves are less toxic than benzoxazinones as discussed in the previous paragraph.

Studies with *O. nubilalis* have also tracked BXD distribution and excretion dynamics using radioactive ^3^H-labeled DIMBOA and MBOA (Campos et al. 1988, 1989). In both cases, the radioactivity was mainly excreted by larvae in the frass and transferred to the pupal case after emergence, suggesting that the adult insect avoids accumulation of BXDs and their metabolites. In fact, a short-term higher level of radioactivity in hemolymph compared to other tissues suggests that these compounds are rapidly transported and excreted. However, the body burden (ratio of radioactivity between body and frass) was constant for all tested concentrations, implying that larvae are not able to increase excretion rate when faced with higher levels of BXDs up to about 2 mM. This is probably consequence of a higher diet consumption, which is consistent with a digestibility-reducing activity of DIMBOA at this concentration. On the other hand, MBOA did not alter diet consumption; thus its effects are due to toxicity and not to antifeedant activity or digestibility reduction.

Further experiments evaluating the effects of DIMBOA and MBOA on food consumption and utilization in *O. nubilalis* also suggest that these compounds have distinct modes of action on insect physiology. Using nutritional indices proposed by Waldbauer (1982), both DIMBOA (0.2 mg/g, ~1 mM) and MBOA (3.0 mg/g, ~18 mM) reduced weight gain in *O. nubilalis* in 4-day artificial diet feeding (Houseman et al. 1992). This was not caused by deterrence of feeding, since the consumption index (CI) increased for DIMBOA and remained the same for MBOA treatments. Instead DIMBOA decreased both approximate digestibility (AD) and efficiency of conversion of ingested food into biomass (ECI), but did not change the efficiency of conversion of digested food into biomass (ECD), indicating that DIMBOA affects digestive processes (e.g. inhibiting digestive enzymes), but does not modify the utilization of nutrients after digestion. In agreement, both in vivo and in vitro assays showed that DIMBOA inhibits trypsin and chymotrypsin. Such reduction in digestibility could lead to the observed increase in diet consumption as an attempt by the larvae to compensate for the decrease in food quality. On the other hand, MBOA ingestion did not change AD or ECI, but decreased ECD, suggesting that this compound has an effect on processes occurring after digestion.

The effects of *O. nubilalis* herbivory on maize metabolism are also relevant for the ecology of this interaction. Larval feeding induced the production of HDMBOA-Glc in stems, with a consequent decrease in DIMBOA-Glc (Dafoe et al. 2011). Even though HDMBOA is considered more toxic than DIMBOA, larvae feeding on previously induced stem sections grew more and consumed more plant tissue than on uninduced stems. Feeding on induced stems decreased CI and AD, but increased ECI and ECD, suggesting that, despite being better defended, such tissues are more nutritive than non-induced stems. Indeed, induction by *O. nubilalis* herbivory increased protein, sucrose, and linoleic acid levels in stems. The authors suggested that high amounts of the auxin 3-indole-acetic acid in *O. nubilalis* oral secretion and frass could affect plant metabolism and promote the increase in nutritional value of attacked tissue.

Given the toxicity and digestibility reducing effects of BXDs on *O. nubilalis*, it may not be surprising that this insect typically feeds on older maize stems (L11–L13 stage) in which the amounts of HDMBOA-Glc and other BXDs are much lower (3.6 µg/g after 48 h induction) than those found in leaves of young seedlings. In contrast, *S. frugiperda* larvae, specialists on maize and other grasses which typically feed on maize at the L4 stage, encounter much higher HDMBOA-Glc concentrations (around 30 µg/g constitutively, and 300 µg/g after herbivore induction in young leaves, their preferred food) (Köhler et al. 2015). Screening of maize lines for resistance towards *O. nubilalis* indicates that in general DIMBOA concentrations of more than 100 µg/g (0.47 mM) in whorl tissues are associated with lower leaf consumption (Barry et al. 1994). Therefore, it has been proposed that *O. nubilalis* restricts its diet to tissues and plant growth stages that contain lower BXD concentrations in order to avoid their toxicity (Maag et al. 2014). This is consistent with the apparently low capability of this insect to detoxify BXDs, as will be discussed in more detail below.

In natural situations, the amount of DIMBOA is not the only factor guiding *O. nubilalis* feeding behavior. In maize plants, *O. nubilalis* larvae prefer to feed on immature whorl tissues, despite their higher BXD levels compared to mature tissues (Bergvinson et al. 1995a, 1995b). Such preference was suggested to be a consequence of higher fiber content and cell wall phenolics in mature tissues, which increase leaf toughness and constitute a mechanical defense towards herbivore feeding. Therefore, it was proposed that neonate larvae feed on the younger, more tender whorl leaves in spite of their higher DIMBOA concentration, because these contain higher amounts of protein. As they grow and develop stronger mandibles better able to chew tougher tissues, the larvae move to more mature parts of the plant with lower levels of BXDs. Another study had revealed a similar trend by comparing maize lines with varying DIMBOA concentrations grown under different light conditions. Even though DIMBOA seems to be responsible for high resistance in some genotypes, neonate leaf consumption and survival correlated better with nitrogen content and nutritional value of plants when comparing different light regimes (Manuwoto and Scriber 1985b).

For the Asian corn borer (*O. furnacalis*), which also feeds extensively on maize as well as other grasses, DIMBOA is also a toxin and antifeedant. Choice assays revealed that DIMBOA applied to cabbage leaves showed increasing antifeedant effects up to 0.8 mg/g (3.79 mM) (Yan et al. 1999). Artificial diets with 1 mg/g DIMBOA (4.73 mM) inhibited growth and extended larval developmental time in this species. However, when compared with *O. scapulalis*, which feeds principally on *Artemisia vulgaris* and less frequently on maize, *O. furnacalis* was less affected by BXDs (Kojima et al. 2010). In *O. furnacalis*, DIMBOA was shown to induce general detoxification reactions including cytochrome P450 and glutathione *S*-transferase activities (Yan et al. 1995). Acetylcholinesterase was inhibited, while inhibition or induction of general esterases depended on the tissue analyzed.

#### Genus *Spodoptera*

Much DIMBOA research has also been carried out on the larvae of another group of lepidopterans, the genus *Spodoptera*, which includes the broad generalist *S. littoralis*, feeding on over 80 families of plants, and the more specialized *S. frugiperda*, which is mostly restricted to feeding on grasses such as maize. As with the *Ostrinia* species discussed above, the larvae of the more specialized *S. frugiperda* perform better on DIMBOA-containing diets than larvae of the generalist *S. littoralis*. When feeding on DIMBOA at 200 µg/g (0.95 mM, the level found in herbivory-induced maize plants), the growth of *S. frugiperda* was similar to that on control diets, but *S. littoralis* grew significantly less (Glauser et al. 2011). However, on diets containing 40 µg/g DIMBOA (0.19 mM), the level encountered in non-induced maize plants, *S. littoralis* did not grow differently than when feeding on a control diet, but *S. frugiperda* grew more quickly. On artificial diets containing 50 and 500 µg/g of the more reactive HDMBOA-Glc, the consumption and preference of *S. littoralis* and *S. frugiperda* did not differ from control diets. However, the addition of maize extract to such diets caused deterrence and lower food intake on the high-HDMBOA-Glc diet for both species (Glauser et al. 2011). This was presumably a consequence of the β-glucosidase activity present in the plant extract, causing hydrolysis of HDMBOA-Glc and releasing the highly toxic HDMBOA aglucone, which persists in the diet for around 30 min. On the other hand, an artificial diet containing the benzoxazolinone MBOA at 330 µg/g (2 mM) did not restrict *S. frugiperda* and *S. littoralis* growth, but decreased the growth of *O. nubilalis* (Maag et al. 2014).

**Fig. 3:**
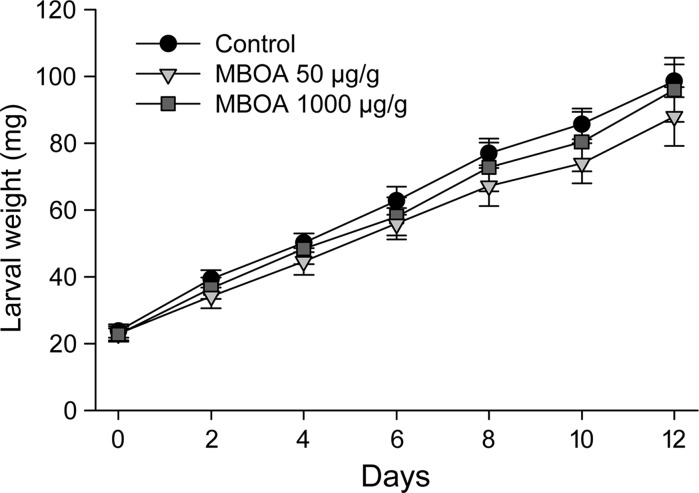
Growth curves (± SEM) for *S. frugiperda* larvae fed on artificial diets containing MBOA

To gain a more detailed understanding of how *S. frugiperda* tolerates BXDs, we recently investigated the effects of MBOA on food consumption and utilization by larvae feeding on bean-based artificial diets by measuring the weights of larvae, diet consumed and frass excreted. MBOA at concentrations of 50 and 1000 µg/g (0.3 and 6.06 mM respectively) did not significantly affect larval growth curves (Fig. 3) or the relative growth rate (RGR) after 12 days of feeding (Table 1) compared to feeding on a control diet lacking MBOA. In the high-MBOA treatment, however, we observed a significant decrease in CI and AD. This seems to be compensated by an increase in both ECI and especially in ECD. Taken together, these data suggest *S. frugiperda* is not affected by MBOA toxicity even at the highest concentration tested, which is higher than the physiological levels encountered in maize seedlings (approximately 2 mM) (Maag et al. 2014). Two hypotheses can be proposed: (1) MBOA exhibits antifeedant activity and decreases CI, but the insect adapts by utilizing its ingested and digested food more efficiently; or (2) MBOA serves as a nutrient for the insect and raises ECD, possibly by increasing nitrogen availability and uptake, with decreased consumption as a response to a richer diet. In any case, MBOA seems to act as a digestibility reducer, either by inhibiting digestive enzymes or interacting with nutrients in the diet and preventing their digestion. The remarkably high effect on ECD indicates that MBOA acts more critically after the digestion process, suggesting a nutrient role. However, *S. frugiperda* has been shown to metabolize MBOA via *N*-glucosylation and to excrete a considerable fraction of it in the frass (Maag et al. 2014). This supports the opposite conclusion, that *S. frugiperda* recruits detoxification enzymes and expends energy (as UDP-glucose) to facilitate MBOA excretion, thus being subject to a higher metabolic cost that should be reflected in a lower ECD. It is important to note that bioassays performed with minimal diets might overestimate the nutritional value of nitrogen-containing compounds and therefore do not necessarily reflect the importance of MBOA in a natural context. Nevertheless, *S. frugiperda* seems to have the capacity of feeding on high MBOA diets without negative effects on growth, and the resulting physiological effects are different than those reported for *O. nubilalis* (Houseman et al. 1992). Although the concentration of MBOA used in this experiment was higher than the concentrations of MBOA usually present in maize, other maize BXDs, such as DIMBOA, DIMBOA-Glc and HDMBOA-Glc, are typically present at mM levels in herbivore-induced plants. Since several BXDs are converted to MBOA in the herbivore gut after hydrolysis (see section below on “Metabolism of BXDs”), herbivores such as *S. frugiperda* could be confronted with MBOA levels similar to those fed in our artificial diets.

**Table 1:** Nutritional indices (±SEM) for *S. frugiperda* larvae fed on artificial diets containing MBOA

Treatment	N	RGR (mg/mg per day)	CI (mg/mg per day)	AD (%)	ECI (%)	ECD (%)
Control	13	0.1055 ± 0.0080	0.9904 ± 0.0530	43.03 ± 3.11	10.83 ± 0.83	27.14 ± 2.88
MBOA 50 µg/g	15	0.0941 ± 0.0055	0.8337 ± 0.0505	39.63 ± 3.41	12.21 ± 1.27	34.98 ± 5.26
MBOA 1000 µg/g	14	0.0989 ± 0.0042	0.6739 ± 0.0387**	27.67 ± 2.83**	15.15 ± 0.85*	66.54 ± 10.02**

* *P* < 0.05, ** *P* < 0.01, Tukey’s test for unequal sample sizes

The feeding preferences of *S. frugiperda* and *S. littoralis* on maize plants at different growth stages parallel the performance differences between the two species (Köhler et al. 2015). While the more specialized *S. frugiperda* preferred to feed on younger leaves in spite of their higher BXD levels and higher degree of induction, feeding of the generalist *S. littoralis* was distributed over different leaf ages, but especially concentrated on older ones. This indicates that *S. littoralis* moved more during foraging, possibly to avoid locally high concentrations of induced BXDs (Maag et al. 2016). After maize stage L6, however, *S. littoralis* switched to young leaves, presumably due to the overall decrease in both BXD levels and inducibility as the plant grows older. Moreover, in mutants containing lower BXD levels, the preference of *S. frugiperda* for young leaves disappeared, and larvae grew less than on high-BXD wild-type plants. This suggests that BXDs differentially influence feeding patterns in *S. frugiperda* (serving as feeding stimulants or nutrients) as compared to *S. littoralis* (acting as deterrents and toxins).

Food consumption and utilization by another generalist *Spodoptera* species, the southern armyworm (*S. eridania*) were compared among maize lines with different DIMBOA levels, which had been bred for resistance towards *O. nubilalis* (Manuwoto and Scriber 1982). Penultimate instar *S. eridania* grew less when feeding on high DIMBOA plants, while showing lower ECD and ECI. These larvae displayed higher CI and AD, even though this was apparently not enough to compensate for the high metabolic costs of feeding on tissue with high DIMBOA levels. However, last instar *S. eridania* larvae grew more on lines with more DIMBOA, possibly because of the induction of detoxification pathways or more efficient food processing caused by previous contact with BXDs. In another study, these authors fed *S. eridania* on two maize genotypes grown under iron and nitrogen deficiencies, which affect water content, and nitrogen and DIMBOA levels (Manuwoto and Scriber 1985a). Overall, Fe-deficient plants showed higher DIMBOA levels, whereas N-deficient plants had lower DIMBOA levels. Surprisingly, larvae feeding on a high-DIMBOA genotype grew more than the group feeding on a low-DIMBOA genotype. Fifth-instar larvae feeding on Fe-deficient plants displayed lower CI and AD, but these were compensated by higher ECD and ECI, resulting in no differences in growth. Nitrogen-deficient plants, however, did not support the development of *S. eridania* as well as controls, resulting in lower growth, CI, ECD, and ECI, together with high mortality rates, even though these plants contained lower DIMBOA levels compared to ones grown with complete nutrient medium. These results suggest that, even though DIMBOA is important in plant resistance to herbivore attack, other factors such as nitrogen and water content also play a role in determining herbivore performance. Deprivation of nutrients might also affect many other primary and secondary metabolites in plant foliage that impact its nutritional value.

In choice assays with another generalist feeding *Spodoptera*, the beet armyworm (*S. exigua*), larval feeding was deterred on barley leaves treated with DIMBOA, while this treatment stimulated feeding for *S. frugiperda* (Rostás 2006). Feeding on artificial diets containing 500 µg/g DIMBOA (2.37 mM) increased mortality and reduced growth in *S. exigua* in short-term experiments, and increased developmental time in long-term experiments, but *S. frugiperda* was not affected. Longer developmental times can affect insect survival in nature making them more vulnerable to predators, parasitoids, and pathogens. However, it is uncertain whether the overall negative effects observed on *S. exigua* are a consequence of DIMBOA toxicity or antifeedant effects.

In summary, benzoxazinones such as DIMBOA seem to be more toxic to caterpillars than benzoxazolinones such as MBOA. The *N*–*O*-methyl derivative HDMBOA is suggested to be even more toxic, also towards BXD-resistant species. Experiments with lepidopteran herbivores indicate that *Spodoptera* spp. are generally more adapted to BXDs than *Ostrinia nubilalis*. And within the genus *Spodoptera*, the more specialized grass feeder *S. frugiperda* seems to be more resistant to the toxic effects of BXDs than *S. littoralis*, *S. eridania*, and *S. exigua*, possibly even benefiting from the presence of BXDs in the diet. Such gradients in BXD resistance are partly explained by the detoxification and metabolic capabilities of each species, as will be discussed in more detail in the following sections. However, insect adaptation to BXD-containing plants might also be influenced by larval feeding behavior and trade-offs between nutritional content and both concentration and induction of chemical defenses (not exclusively BXDs) in plant tissues (McMullen et al. 2009).

### Effects on aphids

Piercing-sucking herbivores from the order Hemiptera, such as aphids, possess modified mouthparts called stylets that are used to pierce through the plant cuticle, epidermis, and mesophyll cells, and feed on the highly nutritious phloem sap (Douglas 2003). Because of this particular feeding behavior, aphids are considered to minimize tissue disruption and consequent activation of glucosylated defenses (Pentzold et al. 2014b). However, the dynamic allocation patterns of BXDs still make them effective defensive compounds towards aphids. Furthermore, apart from their direct toxicity towards aphids, BXDs can trigger callose deposition, serving as a signal to induce another line of plant defense against aphids (Maag et al. 2015a).

BXDs have been considered resistance factors of cereals towards several aphid species. Hydroxamic acid levels in cereals were positively correlated with resistance towards *Metopolophium dirhodium* (Argandoña et al. 1980), *Schizaphis graminum* (Corcuera et al. 1982), and *Sitobion avenae* (Bohidar et al. 1986). Moreover, *Rhopalosiphum padi* displayed higher weight gain and survival when feeding on mutant maize plants with reduced BXD levels compared to wild-type plants (Ahmad et al. 2011). Notably, however, BXD levels in maize were not correlated with resistance towards *R. maidis* (Bing et al. 1990).

The detrimental effects of BXDs on many aphid species have been explored using both artificial diets and plant cultivars with different levels of BXDs. In artificial diets, DIMBOA and MBOA increased mortality in *M. dirhodium* (Argandoña et al. 1980). In *R. padi*, MBOA increased reproduction rate in concentrations up to 0.1 mM, but had the opposite effect above this threshold (Hansen 2006). DIBOA increased *R. padi* mortality in artificial diets, and this was substantiated in a comparison among wild *Hordeum* species containing different DIBOA levels (Barria et al. 1992). DIMBOA also increased *S. graminum* mortality in concentrations as low as 1 mM (Argandoña et al. 1981, 1983) and decreased its reproduction rate in sub-lethal concentrations (0.1 mM) (Corcuera et al. 1982), with DIBOA increasing mortality as effectively as DIMBOA (Zuñiga et al. 1983). In choice assays, DIMBOA had antifeedant activity towards *S. graminum* in artificial diets (Argandoña et al. 1983), whereas *R. padi* avoided wheat leaves from high-DIMBOA cultivars (Givovich and Niemeyer 1991) and barley leaves treated with DIBOA (Copaja et al. 2006). Additionally, aphid species differ in their susceptibility to BXDs. While DIMBOA up to 2 mM in artificial diets increased the mortality of *S. graminum* and *M. dirhodium*, it did not affect *R. maidis* (Corcuera et al. 1982).

Structure–activity relationships comparing the toxicity and antifeedant activity of several BXD aglucones and analogues towards *S. avenae* were determined in artificial diets (Escobar et al. 1999). Among the natural compounds tested, the hydroxamic acids, DIMBOA and DIBOA, elicited remarkably higher mortality (>50 % after 89 h) than the lactams, HMBOA and HBOA (<20 %), and the benzoxazolinones (<10 %). Antifeedant activity and toxicity did not follow the same patterns among the tested compounds. For example, MBOA caused low mortality, but was one of the most deterrent compounds. Such discrepancies indicate that the antifeedant and toxic activities of BXDs do not necessarily arise from the same structural features. In another study, DIBOA and DIMBOA were significantly more repellent to *R. padi* than HBOA and HMBOA were (Bravo et al. 2004). However, in this study BXDs were sprayed on barley leaves, and their allocation and stability during the experiment were not determined.

Interestingly, BXD glucosides are also active towards aphids. In *S. graminum*, DIMBOA and DIMBOA-Glc increased mortality with LD_50_ values (24 h feeding) of 1.2 mM and 4 mM respectively. Both compounds also decreased reproduction rates at concentrations as low as 0.25 mM and caused appreciable feeding deterrence at 0.5 mM (Corcuera et al. 1985). The glucosides DIMBOA-Glc and HDMBOA-Glc increased mortality of *M. dirhodium* with LD_50_ values (3 days feeding) of 5.3 mM and 1 mM, respectively, while also decreasing fecundity. Mortality curves similar to fully unfed treatments suggest that these glucosides are also antifeedant in high concentrations (Cambier et al. 2001). However, it is not clear if BXD glucosides display inherent biological activity towards aphids or are activated (hydrolyzed or otherwise metabolized) by their own enzymes after ingestion.

**Fig. 4:**
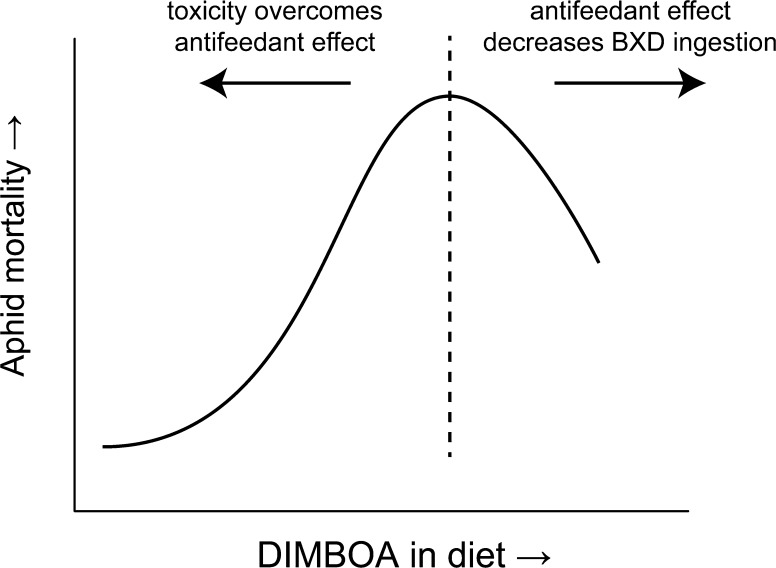
Representation of detrimental effects on aphids caused by different levels of DIMBOA in diet. Above a certain DIMBOA concentration threshold (*dashed line*), the antifeedant effect of DIMBOA overcomes its toxicity, decreasing DIMBOA intake and diminishing mortality in aphids. In higher DIMBOA concentrations, the mortality curves matched those of unfed groups

The lower performance of aphids on high-BXD containing plants likely derives from a combination of toxic and antifeedant effects. Mortality curves for *S. graminum* feeding on artificial diets with 8 mM DIMBOA were similar to a non-fed treatment, suggesting that aphids died due to starvation caused by the antifeedant activity of high DIMBOA concentrations rather than toxicity. In order to separate these two effects, aphids were first exposed to diets containing DIMBOA and then transferred to diets without BXDs (Argandoña et al. 1983). Mortality rates followed a biphasic distribution (illustrated in Fig. 4), being highest at intermediate DIMBOA concentrations (3–4 mM), due to the toxicity of DIMBOA ingested in the first diet. Mortality decreased at higher DIMBOA concentrations due to lower diet ingestion and DIMBOA uptake, caused by its strong antifeedant effect. A similar experiment comparing DIMBOA and DIMBOA-Glc resulted in highest mortalities at 4 mM and 6 mM respectively (Corcuera et al. 1985). Upon feeding on wheat with different DIMBOA contents, the same biphasic profile was observed for DIMBOA content in bodies of *M. dirhodium* and *S. avenae* (Niemeyer et al. 1989), and for both honeydew production and DIMBOA-Glc content in honeydew of *R. padi* (Givovich et al. 1992), which are parameters reflecting food ingestion.

BXDs might increase aphid susceptibility to other plant chemical defenses and insecticides by decreasing their detoxification capabilities. In *R. padi*, DIMBOA inhibits glutathione-*S*-transferases and esterases in vivo at dietary concentrations as low as 1.0 and 0.5 mM, respectively (Mukanganyama et al. 2003). UGT activities in *S. avenae* were lower when the aphids fed on high-DIMBOA wheat cultivars than on low-DIMBOA cultivars, and UGT inhibition by DIMBOA was confirmed in vitro (Leszczynski et al. 1992).

The effect of BXDs on aphids depends on their abundance in phloem and other tissues penetrated by these insects on their way to the phloem. In wheat, DIMBOA-Glc was the only BXD hydroxamic acid in phloem sap present at concentrations around 1 mM. Average DIMBOA-Glc concentrations in the phloem did not differ greatly among cultivars with different BXD levels. However, a high variability of DIMBOA-Glc levels was found among phloem samples from the same cultivar which may reflect different BXD biosynthesis or transport activity in particular sieve tubes, and suggests that aphids could choose sieve tubes according to their BXD levels (Givovich et al. 1994). Among other tissues, BXD hydroxamic acids were also detected in the mesophyll, but not in the xylem in maize and wheat leaves (Argandoña and Corcuera 1985, Argandoña et al. 1987). Furthermore, the aglucone DIMBOA and the glucosides DIMBOA-Glc and HDMBOA-Glc were present in the leaf apoplast in maize (Ahmad et al. 2011) and so might be perceived during aphid stylet penetration. That aphids do encounter BXDs at all is demonstrated not only by their negative effects on aphid performance in the whole plant bioassays as cited above, but also by the presence of these compounds in honeydew. DIMBOA-Glc was detected in honeydew of *R. padi* (Givovich et al. 1992) and *S. avenae* (Leszczynski and Dixon 1990) feeding on wheat seedlings, but DIMBOA aglucone and MBOA were low or completely absent.

Due to the feeding behavior of aphids, a compound present in the phloem sap can exert both antifeedant and toxic effects, while compounds located in the path followed to reach the phloem are considered to have only antifeedant effects (Cambier et al. 2001) since aphids do not ingest such encountered compounds at a high rate. In this context, the electrical penetration graphic (EPG) technique (Tjallingii 1985) allows the evaluation of individual parameters of overall aphid probing behavior and their modification by BXDs. As the hydroxamic acid content of wheat cultivars increases, *S. graminum*, *R. padi*, *S. avenae*, and *M. dirhodium* took longer to begin phloem ingestion, and exhibited increased xylem ingestion, possibly to “dilute” ingested BXDs (Givovich and Niemeyer 1991; Givovich et al. 1994). On the other hand, for *R. maidis*, the time to reach the phloem was not influenced by the plant BXD content, suggesting that this species is insensitive to the antifeedant effect of BXDs while searching for sieve elements. Furthermore, the duration of phloem ingestion did not change according to BXD levels in the plant for any of these five species, implying that BXDs are more critical before the phloem is reached. Surprisingly, DIMBOA and DIMBOA-Glc offered in artificial diets decreased ingestion time for all five aphid species tested, including *R. maidis*, indicating that both compounds have a similar dose-dependent antifeedant activity on all species in this context. The feeding strategy adopted by *R. maidis* consists in puncturing fewer cells before reaching the phloem than *R. padi*, as indicated by EPG comparisons. Therefore, *R. maidis* minimizes exposure to BXDs in mesophyll cells and avoids their antifeedant effects, although it still suffers from them when BXDs are administered via artificial diets (Givovich and Niemeyer 1995). The influence of BXDs on the feeding behavior of *Diuraphis noxia* is similar to that on the other species described above (Givovich and Niemeyer 1996; Mayoral et al. 1996).

Avoidance of BXDs might be learned by previous experience, as shown by *Sitobion fragariae* aphids feeding on wheat cultivars with different BXD levels (Ramirez et al. 1999). In a high-BXD cultivar, the time taken by naïve aphids to reach the phloem was higher than in a low-BXD cultivar. However, on a second probing, the time to achieve phloem ingestion did not differ among cultivars. A similar trend was observed for the number of mesophyll cell punctures. Such effects were also observed upon feeding on attacked and non-attacked plants, indicating that no aphid-induced effects in the plant are involved. These data suggest that aphids can adapt their feeding behavior after exposure to BXDs to avoid them in future probes by minimizing mesophyll cell damage.

In summary, these data suggest that BXDs protect plants against aphid attacks, acting at different stages from probing to feeding. Before probing has started, aphids can potentially already sense BXD aglucones such as HDMBOA present in leaf surface waxes. Once the epidermis is penetrated, the stylet passes mostly through the apoplast, where BXD glucosides are present. On its way to the phloem, the aphid punctures and probes mesophyll cells, possibly with mechanical disruption of organelles (Brzezina et al. 1986; Hewer et al. 2011). Such a scenario could lead to hydrolysis of BXD glucosides by plant β-glucosidases and expose the aphid to locally high concentrations of antifeedant BXD aglucones. Even if BXD hydrolysis does not occur, the intact glucosides appear to exert antifeedant activity as well. However, it is not clear whether BXD aglucones and glucosides present in the apoplast are perceived by aphid chemoreceptors and provoke antifeedant responses in this way. Finally, once the aphid reaches a suitable sieve element and starts feeding on the phloem sap, it ingests considerable amounts of BXDs, mostly glucosides. However, the duration of phloem ingestion does not seem to correlate to BXD content in plants, which suggests that BXDs in phloem sap are not concentrated enough to exert antifeedant effects or are masked by other phloem constituents.

The antifeedant effects of BXDs towards aphids may not necessarily arise from the same BXDs implicated as antifeedants or toxins against chewing herbivores. Thus, when selecting insect resistant cereal lines, more than one BXD metabolite may be required to cover the full spectrum of insect pests. On the other hand, due to the dependence of aphids on bacterial endosymbionts, the antimicrobial activities of BXDs could also contribute to their overall detrimental effects on aphids. Future investigations on the effects of BXDs on aphids would benefit from taking into account their specialized feeding behavior. Studies using artificial diets offer BXDs as a homogeneous solution and so do not account for their specific allocation to leaves and possible aphid avoidance behavior during probing. Although artificial diet bioassays are useful to assess BXD toxicity, they might overestimate antifeedant effects. Alternatively, EPG studies are useful to determine what features of aphid feeding behavior are associated with BXD avoidance, and how insects respond to them, especially insensitive species such as *R. maidis*.

BXDs may also impact maize defense against aphids by serving as a signal for callose deposition. Maize mutants deficient in BXD production showed lower callose deposition when treated with chitosan, a well-known elicitor of defense responses to fungal pathogens, compared to BXD producing lines (Ahmad et al. 2011). Furthermore, DIMBOA (but not HDMBOA-Glc) induced callose deposition when infiltrated into maize leaves. In another study, callose deposition induced by *R. maidis* feeding was lower on maize lines with low DIMBOA-Glc (and high HDMBOA-Glc) content, and aphid performance was better, even though HDMBOA-Glc is more toxic to aphids than DIMBOA-Glc when administered in artificial diets (Meihls et al. 2013). Experiments with maize recombinant inbred lines also reported a negative correlation between *R. maidis* reproduction and DIMBOA content and callose formation (Betsiashvili et al. 2015). However, it is difficult to separate the direct detrimental effects of BXDs on aphids (antifeedant and toxic effects) from indirect effects due to their signaling roles (callose deposition), and further detailed experiments are required to investigate such relations. Both of these types of effects likely play a role in overall plant resistance to insect herbivores, but it is also possible that individual herbivore species are distinctly affected, as observed for *R. maidis*, which seems to be insensitive to the direct antifeedant effects of BXDs, as discussed above, but is susceptible to the induction of callose.

### Effects on root herbivores

Insect herbivores feeding on belowground plant tissues have had to adjust to the challenges posed by living in the soil and the specific primary and secondary metabolites of roots. Root herbivores are influenced by the antifeedant and toxic activities of plant chemical defenses and exploit root volatiles and exudates for host location and foraging similarly to aboveground herbivores (Erb et al. 2013; Hiltpold et al. 2013). Although progress is being made in this field, the release of BXDs and other secondary metabolites by roots is still not well understood (Baetz and Martinoia 2014; Park et al. 2004).

The effect of BXDs on the Western corn rootworm (*Diabrotica virgifera virgifera*) has been widely studied due to its economic importance as a specialist maize pest. DIMBOA applied to corn roots increased mortality of *D. v. virgifera*, but it is not clear how DIMBOA was absorbed and possibly metabolized by maize roots and the actual concentrations experienced by the insects. At the highest concentrations applied, larvae came out of the roots before dying, suggesting an antifeedant effect and death by starvation. However, larval death was also observed inside the root, indicating that toxicity also contributes to high mortality. Upon infestation with *D. v. virgifera* eggs, a high-DIMBOA maize line (1,300 µg/g root fresh weight, 6.16 mM) suffered less damage than a low-DIMBOA line (400 µg/g, 1.90 mM). Insect infestation in the high-DIMBOA line led to a lower adult emergence rate and size when compared to the low-DIMBOA line (Xie et al. 1990). In another experiment, *D. v. virgifera* feeding on maize lines with root DIMBOA levels ranging from 90 to 250 µg/g (0.43–1.18 mM) did not differ in developmental time and survival, confirming that these values are below a threshold for resistance against this herbivore (Davis et al. 2000). Nevertheless, the positive correlation between root BXD content and resistance towards *D. v. virgifera* damage was also observed in field experiments with different maize lines (Assabgui et al. 1995a, b).

When applied to maize roots, DIMBOA, DIBOA, DIM_2_BOA, HMBOA, and MBOA were repellent to *D. v. virgifera* larvae, as observed by choice assays (Xie et al. 1992). Fewer larvae were found inside roots and more stayed on or outside roots when compared to control treatments. BXD treatment also modified host searching behavior, decreasing number of turns and increasing area searched and the locomotion of larvae. A first bioassay-driven fractionation of maize root extracts suggested that MBOA is attractive to *D. v. virgifera* and could be used as a volatile chemical cue for location of grass hosts in a CO_2_-rich background (Bjostad and Hibbard 1992). However, in a second study, the authors found CO_2_ to be the only compound responsible for larval attraction, rather than other components of maize extracts (Bernklau and Bjostad 1998). Furthermore, MBOA applied to maize roots did not show antifeedant activity or toxicity towards *D. v. virgifera* (Abou-Fakhr et al. 1994).

However, recent studies indicate that *D. v. virgifera* uses BXDs as chemical cues for foraging, despite their toxicity. Maize crown roots were shown to be more nutritious and also to contain higher levels of total and exuded BXDs than primary roots. Nevertheless, *D. v. virgifera* preferred to feed on crown roots, while feeding by the generalist *D. balteata* was more distributed between crown, primary, and secondary roots. Accordingly, in no-choice assays, *D. v. virgifera* larvae gained more weight feeding on crown roots compared to primary roots in both wild-type and low-BXD mutant plants. However, when given the choice between crown and primary roots, *D. v. virgifera* did not show any preference in low-BXD mutants, suggesting that this insect uses BXDs as chemical cues to locate highly nutritious roots (Robert et al. 2012).

The performance of *D. v. virgifera* was compared to the generalist southern corn rootworm (*Diabrotica undecimpunctata howardi*) when feeding on a BXD-deficient mutant with low BXD levels and its parental line with high BXD levels (Alouw and Miller 2015). Survival and developmental time were the same when comparing both maize lines. However, *D. v. virgifera* grew better on the high-BXD line compared to the low-BXD mutant, while no differences were observed in *D. u. howardi* growth. The better performance of the specialist *D. v. virgifera* might be related to its ability to exploit BXDs to find nutritious tissues (Robert et al. 2012), while the generalist *D. u. howardi* is unable to do so. The results from Robert et al. (2012) and Alouw and Miller (2015) suggest that *D. v. virgifera* is tolerant to BXDs and seem to contradict previous studies showing strong antifeedant and toxic effects. It is possible that by feeding on tissues with high nutritional value, *D. v. virgifera* overcomes the detrimental effects of BXDs, resulting in better overall performance. On the other hand, the application of pure BXDs onto the root surface used to show repellency in earlier studies does not necessarily reproduce the insect experience in a natural context, considering that their absorption and metabolism by the plant and potential effects of other co-exuded compounds and enzymes are not well-known. Additionally, the earlier studies comparing maize lines only focused on DIMBOA content, not considering other BXDs (such as HDMBOA-Glc) and their distribution among root tissues, which might also play an important role in feeding preference and toxicity against *D. v. virgifera*.

Another specialized root herbivore, the wheat bulb fly (*Delia coarctata*), showed remarkable attraction to wheat seedling exudates (Rogers and Evans 2013). MBOA attracted larvae in a dose-dependent fashion and might contribute to the activity of the exudate, while DIMBOA elicited a weaker response. Since MBOA is more stable than DIMBOA in the soil (Macías et al. 2004), it constitutes a more reliable chemical cue for host location.

In soil, BXDs and their derivatives are present as a complex and dynamic mixture whose composition depends on many factors such as temperature, pH, and soil microbiota. Moreover, soil fungi are known to metabolize benzoxazolinones and produce a variety of aminophenols and aminophenoxazinones, as well as their malonylated and acetylated derivatives (Fomsgaard et al. 2004). Such compounds could also possess biologically relevant activities on root herbivores, but this has not yet been investigated in detail. Soil nematodes and microbial pathogens are also exposed to BXD profiles in the soil environment and are subject to their biological activities (Meyer et al. 2009; Zasada et al. 2005). Due to the complexity of the soil matrix, it is difficult to design bioassays that reflect the BXD concentrations and allocation in the root tissue as perceived by a root herbivore in a natural context. Further studies on BXD degradation and diffusion rates through soil could furnish information to construct more realistic experimental setups to evaluate BXD influence on underground plant protection.

## Metabolism of BXDs

Despite the toxicity, allelopathic and antimicrobial activities of BXDs, some animals, plants, and microorganisms are able to avoid these detrimental effects. Recent reports have increased our understanding about how BXDs are metabolized, absorbed, and excreted by target organisms. Such knowledge provides insight into the coevolution of these plant chemical defenses with plant enemies and the effects of cereal products on human health.

### Metabolism in insects

**Fig. 5:**
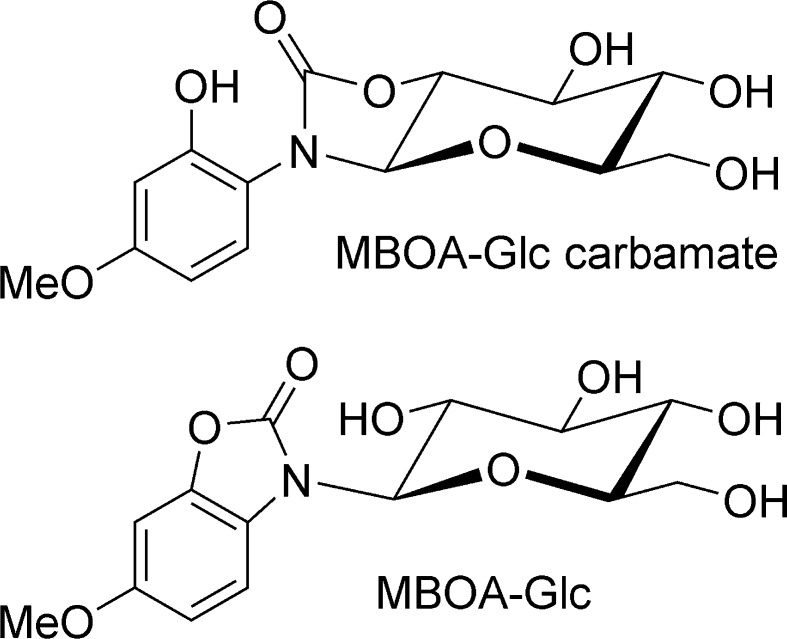
Structures of MBOA metabolites detected in plants and insect frass

Several insect species use BXD-containing plants as food, which suggests they have developed resistance strategies such as avoidance, rapid excretion, sequestration, detoxification, or target-site mutation (Després et al. 2007). For example, larvae of *M. separata* fed on artificial diet containing DIMBOA were found to excrete DIMBOA-Glc, HMBOA-Glc and 1-(2-hydroxy-4-methoxyphenylamino)-1-deoxy-β-glucopyranoside-1,2-carbamate (referred to here as MBOA-Glc-carbamate, Fig. 5) in the frass based on NMR analyses (Sasai et al. 2009) in accordance with previously published data (Hofmann et al. 2006; Sicker et al. 2001). Incubation of midgut homogenates with DIMBOA and UDP-glucose yielded DIMBOA-Glc, indicating a UGT activity. The in vitro DIMBOA glucosylation activity for *M. separata* was higher than for the non-adapted *B. mori*.

Metabolism of DIMBOA is also observed upon incubation of *O. furnacalis* larval gut homogenates with UDP-glucose (Kojima et al. 2010). This conversion is not affected by the presence of NADPH or glutathione, and is decreased if the homogenate is treated with heat or proteinases, confirming its enzymatic nature. Assays with larvae of *O. scapulalis* and *O. latipennis*, two species known to be less well-adapted than *O. furnacalis* to feed on BXD-containing plants, resulted in lower DIMBOA conversion rates. DIMBOA metabolism by *O. furnacalis* larval gut homogenates had a pH optimum between 7.2 and 7.8 and was induced after feeding on DIMBOA-containing diet or maize, which was not observed for *O. scapulalis* (Phuong et al. 2015). However, DIMBOA-Glc was not detected, and other products were not identified.

Other species that are well adapted to feeding on BXD-containing plants, such as *S. frugiperda*, have also been suggested to have resistance mechanisms towards these compounds. Both *S. frugiperda* and *S. littoralis* larvae feeding on DIMBOA-containing diet were shown to diminish its toxicity by glucosylation reactions, excreting DIMBOA-Glc, HMBOA-Glc, and MBOA-Glc in the frass (Glauser et al. 2011). On the other hand, HDMBOA is considered to be too unstable (Maresh et al. 2006) in the alkaline insect gut to be efficiently conjugated, in agreement with its potent toxic and antifeedant effect even towards the BXD-resistant *S. frugiperda*. This instability may prevent reglucosylation even if a glucosyltransferase with the appropriate substrate specificity were present.

In a screening of many lepidopteran species, glucosylation was shown to be an important process in BXD metabolism. When feeding on maize leaves, larvae of the more resistant species, *S. frugiperda*, *S. littoralis*, and *S. exigua*, excreted DIMBOA-Glc in the frass, while the more susceptible *Mamestra brassicae* and *Helicoverpa armigera* did not (Wouters et al. 2014). *In vitro* assays confirmed that DIMBOA is glucosylated by *S. frugiperda* larval gut homogenates in the presence of UDP-glucose, suggesting the contribution of UGT enzymes. The insect-derived product, (2*S*)-DIMBOA-Glc, is an epimer of the original (2*R*)-DIMBOA-Glc produced by the plant. Such a change in stereochemistry results in the glucoside no longer being a substrate recognized by the plant BXD β-glucosidases, which are still present and active in the gut lumen. Thus this transformation represents a detoxification mechanism since the toxic aglucones can no longer be formed.

**Fig. 6:**
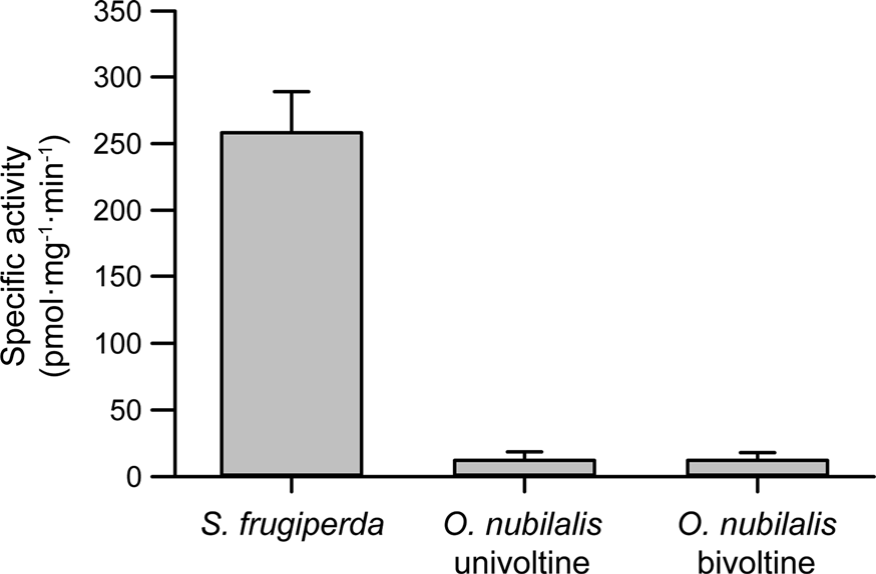
Specific activity of MBOA glucosylation (±SEM) in gut homogenates of *S. frugiperda* and *O. nubilalis* (univoltine and bivoltine strains) (N = 3)

The benzoxazolinone MBOA is also glucosylated by lepidopteran herbivores. This compound is a product of the spontaneous degradation of DIMBOA and HDMBOA (Fig. 1), two of the most abundant BXDs present in maize leaves, but also shows its own inherent toxicity. Since the alkaline gut of most lepidopteran larvae accelerates DIMBOA and HDMBOA degradation to MBOA, insects are exposed to high levels of this BXD. *S. frugiperda* and *S. littoralis* larvae feeding on artificial diets containing MBOA excreted considerable amounts of MBOA-Glc, while *O. nubilalis* larvae were less efficient in this conjugation reaction (Maag et al. 2014). Detailed structural elucidation revealed that the observed MBOA metabolite was 3-β-D-glucopyranosyl-6-methoxy-2-benzoxazolinone (MBOA-Glc, Fig. 5) rather than the isomeric MBOA-Glc-carbamate previously characterized as a plant detoxification product (Hofmann et al. 2006; Sicker et al. 2001). To quantify these transformations more directly, we compared the rate of MBOA glucosylation by UDP-glucosyltransferases using in vitro assays of gut homogenates of *S. frugiperda* and *O. nubilalis* larvae. *S. frugiperda* displayed a specific activity for MBOA more than 20-fold higher than *O. nubilalis*, and no significant differences were observed between *O. nubilalis* univoltine and bivoltine strains (Fig. 6). Accordingly, the tolerance of *O. nubilalis* to BXD-containing plants appears not to depend on BXD detoxification, but rather on temporal aspects and foraging preferences (Maag et al. 2014). However, it is not known whether UGT activity towards BXDs in *O. nubilalis* is induced upon exposure to BXDs. The regulation and identity of the UGTs responsible for BXD detoxification in *S. frugiperda* are currently being investigated.

A recent comparison between the performance and transcriptional profiles of *S. frugiperda* and *S. littoralis* showed that the total expression of UGT-encoding genes did not change between larvae feeding on artificial diet and on maize leaves (Roy et al. 2016). This supports the hypothesis that the enzymes responsible for BXD glucosylation are constitutively expressed in *Spodoptera* spp., rather than being induced upon contact with BXDs. Furthermore, *S. frugiperda* performance on maize was higher than that of *S. littoralis*, which indicates a greater tolerance to BXDs.

To the best of our knowledge there are no detailed studies investigating the metabolism of BXDs in aphids. However, *S. avenae* reared on wheat over 10 generations possessed increased activities of cytochrome P450 monooxygenases, NADPH cytochrome *c* reductases, glutathione *S*-transferases, and esterases, but not catalases (Loayza-Muro et al. 2000). In general, these increases were more pronounced in the low-BXD wheat cultivar tested, presumably due to antifeedant activity and limited BXD ingestion by aphids reared on the high-BXD cultivar. Such upregulation of detoxification enzymes might indicate they are involved in BXD metabolism, but more studies are necessary to confirm this hypothesis. Aphids also have a wide range of enzymes in their saliva (Madhusudhan et al. 1994), which can potentially be secreted during probing and modify BXDs, as has been proposed for phenols (Urbanska et al. 1998).

Tolerance towards BXDs has also been suggested for *D. v. virgifera*. The transcriptional profiles of larvae feeding on a low-BXD maize mutant and on the high-BXD parent line were compared (Miller and Zhao 2015). Differentially expressed genes included a cytochrome P450 and a cathepsin protease. This indicates that *D. v. virgifera* might detoxify BXDs using P450s, but this hypothesis remains to be tested.

Besides detoxification, other strategies such as behavioral avoidance and sequestration might contribute to the resistance towards BXDs observed in some insect species. For example, the act of snipping the leaf into larger pieces during feeding minimizes plant tissue disruption and, together with an alkaline gut, can inhibit the activation of plant chemical defenses such as BXDs. These mechanisms allow *Zygaena filipendulae* to limit hydrolysis of cyanogenic glucosides in its host plant and to sequester these compounds for use against predators (Pentzold et al. 2014a).

### Metabolism in mammals

In addition to their importance in assessing the safety and therapeutical uses of BXD derivatives, the metabolism of BXDs in mammals may reveal some common strategies that other animals, including insects, employ to metabolize these compounds. Ingestion of rye bread which naturally contains a mixture of BXDs has revealed some aspects of BXD metabolism in mammals. For example, in rats and pigs, glucoside hydrolysis, hydroxamic acid reduction to the corresponding lactam, and aglucone conjugation to glucuronic acid were important metabolic reactions (Adhikari et al. 2012a, b). In humans, hydroxamic acid reduction and aglucone conjugation with glucuronic acid and sulfate were observed (Adhikari et al. 2013). However, a considerable part of the ingested BXDs was not recovered in the urine and feces in the three studies, indicating that absorption or transformation to unknown metabolites also took place.

## Conclusions and suggestions for future research

Benzoxazinoids constitute a class of activated plant defenses that function against a wide range of insect herbivores and other target organisms. In this review, we have seen that the effects of BXDs on different guilds of insect herbivores are strongly influenced by the specific distributions of the compounds themselves and their activating β-glucosidases. BXD effects are also determined by the unique structural features of these molecules which give them different reactivities, with benzoxazinone *N*-substitution with either –H, –OH or –OCH_3_ in particular giving rise to very contrasting aglucone characteristics. Such chemical variation helps to diversify and promote these compounds’ modes of action as toxins, feeding deterrents and digestibility-reducing compounds. Plants can take advantage of such chemical diversity by specifically inducing *de novo* BXD biosynthesis and regulating their interconversion and accumulation in specific tissues, adapting their defensive strategy to the nature of the attacking insect herbivore. As we have also seen, insect species have varying susceptibility to BXDs not only arising from these bioactivities, but resulting also in part from their ability to metabolize these substances into less harmful derivatives, as well as the distribution and induction of individual BXDs in leaves of different ages and nutritional backgrounds.

Herbivore feeding and performance assays on artificial diets are important tools to assess the effects of individual BXDs in a dose-dependent way. However, the stability of these compounds under the bioassay conditions must be considered. Like derivatives of other activated defense compounds, BXD hydrolysis products can react with other diet components (Argandoña et al. 1982) or degrade to other substances such as benzoxazolinones, which might modify the final results. For example, most HDMBOA in an artificial diet degraded within 30 min after HDMBOA-Glc hydrolysis (Glauser et al. 2011) and a similar, albeit slower, decomposition was observed for DIMBOA (Campos et al. 1989). As the degradation of BXD hydroxamic acids and *N*–*O*-methyl derivatives to benzoxazolinones is faster at high pH values (Maresh et al. 2006; Niemeyer et al. 1982a), the acidification of diets constitutes an alternative to improve BXD stability during feeding bioassays (Argandoña et al. 1982). Similarly, BXDs applied on plant leaves for bioassays can also degrade or be metabolized quickly, and their persistence should be assessed in such experiments. Additionally, the BXD defense system in plants, like that of other activated defenses, is compartmentalized and depends on temporally and spatially resolved activation by hydrolysis. A bioassay that simulates the tissue-specific distribution of BXDs present in a plant leaf or the gradients of BXDs diffusing through soil would be very valuable, but difficult to design. In the meantime, the limitations of existing experimental bioassay setups must be kept in mind.

Studies on the influence of BXDs on food consumption and utilization by insect herbivores are useful to discriminate toxins, digestibility-reduction agents, and antifeedant factors. However, such bioassays should take into account that the concentration and composition of plant BXDs, like that of many other defense compounds, are rapidly and locally induced by the act of herbivory, and in the insect detoxification genes are induced by initial contact with BXDs. Artificial diets provide a more controlled way to assess the effects of single compounds on insect physiology, but have limitations in representing the plant, since induced responses are absent and the nutritional composition of diets is different than the plant, which may modulate toxin activities (Duffey and Stout 1996). In bioassays using specific BXD mutants, the nutritional values and levels of non-BXD metabolites in different lines and plant tissues should also be considered as these may have changed in unplanned ways.

For studies on BXD metabolism by insects and other target organisms, future work in determining the structures of metabolites and quantifying their abundance will benefit from ongoing advances in the sensitivity and resolution of modern analytical instruments. In addition, the development of mutant plant lines with low levels of BXDs facilitates the identification of new metabolites by comparative metabolomic approaches (Glauser et al. 2011; Maag et al. 2015b). Nevertheless, feeding single compounds via artificial diets may still be necessary to identify individual metabolites, especially considering the often interlinked metabolism of BXDs, with conversion of hydroxamic acids to lactams, and of multiple benzoxazinones to single benzoxazolinone derivatives. The structural elucidation of metabolites gives rise to hypotheses about the enzymatic pathways involved, which can be confirmed in vitro and characterized on the gene level.

The toxicokinetics of BXDs is another important aspect in the description of their biological activities in insects. The absorption, distribution, and excretion of BXDs can be followed with high sensitivity by using radioactive isotopically labelled compounds (Campos et al. 1988, 1989), but these substances are not readily available. Careful studies with unlabeled compounds can also provide important insights on physiological processes and suggest the involvement and location of transporters and detoxification enzymes, and other strategies such as sequestration.

Further understanding of the biological effects of BXDs on target organisms and their metabolism requires the convergence of many disciplines, including ecology, evolutionary biology, biochemistry, analytical chemistry, and organic synthesis. The resulting knowledge will undoubtedly provide many new insights on the interactions between BXD-containing plants and other organisms, and can also contribute to the development of new strategies for pest control on BXD-containing crops, such as wheat and maize, that lead to the breeding of new resistant varieties or the synthesis of novel compounds which alter insect BXD processing.

## Materials and methods

### Insects

Larvae of *S. frugiperda* (maize strain) were a generous gift from the Department of Entomology of the Max Planck Institute for Chemical Ecology, and were reared on an artificial diet based on white beans (Bergomaz and Boppré 1986), under controlled light and temperature conditions (12:12 h light/dark, 20 °C). Eggs from univoltine and bivoltine strains of *O. nubilalis* were obtained from the Agroscope Changins (Switzerland) and were reared under the same conditions described above. The *O. nubilalis* diet was adapted from the literature (Maag et al. 2014), using barley flour instead of wheat germ.

### Nutritional indices

Third instar larvae were individually kept on plastic cups and under the rearing conditions described above for 12 days. MBOA was added to the diets during the cooling step of the preparation process as a solution in ethanol (5 mL for each 100 g diet) or pure ethanol for the control treatment. The diets were poured into Petri dishes, and diet plugs were cut with a cork borer, left for 15 min for ethanol evaporation, and offered to the insects. Diet plugs were replaced daily. Frass and remaining diet in cups were collected daily, freeze-dried overnight, and weighed in order to quantify ingested food and feces. Larvae were weighed every second day to assess growth. At the end of the experiment and at the end of the life stages studied, larvae were freeze dried and shown to have consistent water content (85 %). All larval weights were calculated as dry mass using water content of each individual at the end of the experiment. Overall weight gain, mean weight, ingested food, and feces were calculated in terms of dry mass over the 12 days of the experiment, and nutritional indices were calculated according to the following equations (Waldbauer 1982):

$$\begin{aligned}{\text{RGR}} &= \frac{\text{Weight gained}}{{{\text{Time }} \times {\text{Mean weight}}}}\\ {\text{CI}} &= \frac{\text{Ingested food}}{{{\text{Time }} \times {\text{Mean weight}}}}\\ {\text{AD}} &= \frac{{{\text{Ingested food }} - {\text{Feces}}}}{\text{Ingested food}} \times 100 \\ {\text{ECI}} &= \frac{\text{Weight gained}}{\text{Ingested food}} \times 100\\ {\text{ECD}} &= \frac{\text{Weight gained}}{{{\text{Ingested food }} - {\text{Feces}}}} \times 100\end{aligned}$$

### Enzymatic assays

Gut homogenates for in vitro assays were dissected from third and fourth instar larvae of *S. frugiperda* and *O. nubilalis*. The caterpillars were dissected in cold 10 mM phosphate buffer (pH 7.0) and the gut tissue was isolated and its contents removed. The rinsed gut tissues were then transferred to a fresh tube and homogenized with 100 µL of 10 mM phosphate buffer (pH 7.0) per gut. Protein concentrations were determined using the method of Bradford (1976). For the in vitro assays, 10 µL of gut suspension were incubated with 75 nmol of MBOA and 150 nmol of UDP-glucose in 100 mM phosphate buffer at pH 7.0 (final assay volume: 50 µL). After an incubation period of 60 min at 30 °C the reaction was stopped by adding 50 µL of MeOH/formic acid (50:50, v/v). The samples were centrifuged at 5000 g for 5 min prior to analysis by HPLC–MS/MS. Aliquots of the gut homogenates were heated at 100 °C for 15 min and used for boiled controls.

### Chromatographic methods

For analytical chromatography procedures, formic acid (0.05 %) in water and acetonitrile were used as mobile phases A and B, respectively, and the column temperature was maintained at 25 °C. The quantitative analysis of MBOA-Glc produced in in vitro assays used an XDB-C18 column (50 × 4.6 mm, 1.8 µm, Agilent Technologies, Boeblingen, Germany) with a flow rate of 1.1 mL min^−1^ and with the following elution profile: 0–0.5 min, 95 % A; 0.5–6 min, 95–67.5 % A; 6.02–7 min, 100 % B; 7.1–9.5 min, 95 % A. HPLC–MS/MS analyses were performed on an Agilent 1200 HPLC system (Agilent Technologies, Boeblingen, Germany) coupled to an API 3200 triple quadrupole mass spectrometer (Applied Biosystems, Darmstadt, Germany) equipped with a turbospray ion source operating in negative ionization mode. The ion spray voltage was maintained at −4500 V. The turbo gas temperature was 500 °C, nebulizing gas 60 psi, curtain gas 25 psi, heating gas 60 psi and collision gas 5 psi. Multiple reaction monitoring (MRM) was used to monitor the analyte parent ion to product ion conversion with MRM parameters for MBOA-Glc optimized from infusion experiments with a standard (Q1 *m/z*: 372, Q3 *m/z*: 164, DP −15 V, EP −4.5 V, CEP −18 V, CE −20 V, CXP −4 V). Both Q1 and Q3 quadrupoles were maintained at unit resolution. Analyst 1.5 software (Applied Biosystems, Darmstadt, Germany) was used for data acquisition and processing.
